# Citation classics in central nervous system inflammatory demyelinating disease

**DOI:** 10.1002/brb3.700

**Published:** 2017-04-19

**Authors:** Jee‐Eun Kim, Kang M. Park, Yerim Kim, Dae Y. Yoon, Jong S. Bae

**Affiliations:** ^1^ Department of NeurologySeoul Medical CenterSeoulKorea; ^2^ Department of NeurologyHaeundae Paik HospitalInje University College of MedicineBusanKorea; ^3^ Department of NeurologyKangdong Sacred Heart HospitalHallym University College of MedicineSeoulKorea; ^4^ Department of RadiologyKangdong Sacred Heart HospitalHallym University College of MedicineSeoulKorea

**Keywords:** bibliometrics, central nervous system inflammatory demyelinating disease, citation analysis, multiple sclerosis, neuromyelitis optica

## Abstract

**Objectives:**

To identify and analyze the characteristics of the most influential articles about central nervous system (CNS) inflammatory demyelinating disease.

**Materials and Methods:**

The Institute for Scientific Information (ISI) Web of Science database and the 2014 Journal Citation Reports Science Edition were used to retrieve the top 100 cited articles on CNS inflammatory demyelinating disease. The citation numbers, journals, years of publication, authorships, article types, subjects and main issues were analyzed. For neuromyelitis optica (NMO), articles that were cited more than 100 times were regarded as a citation classic and described separately.

**Results:**

The top 100 cited articles were published between 1972 and 2011 in 13 journals. The highest number of articles (*n* = 24) was published in *Brain,* followed by *The New England Journal of Medicine* (*n* = 21). The average number of citations was 664 (range 330–3,897), and 64% of the articles were from the United States and the United Kingdom. The majority of the top 100 cited articles were related to multiple sclerosis (*n* = 87), and only a few articles reported on other topics such as NMO (*n* = 9), acute disseminated encephalomyelitis (*n* = 2) and optic neuritis (*n* = 2). Among the top 100 cited articles, 77% were original articles. Forty‐one citation classics were found for NMO.

**Conclusions:**

Our study provides a historical perspective on the research progress on CNS inflammatory demyelinating disease and may serve as a guide for important advances and trends in the field for associated researchers.

## 1 Introduction

Central nervous system inflammatory demyelinating disease (CIDD) is a term that encompasses a broad spectrum of diseases such as multiple sclerosis (MS), neuromyelitis optica (NMO), acute disseminated encephalomyelitis (ADEM), optic neuritis (ON), and transverse myelitis. MS is a prototypic form of CIDD that has garnered great interest by researchers worldwide due to its high prevalence, young age of onset and chronicity, which results in a significant social burden (Adelman, Rane, & Villa, 2013). Recent understanding of the role of aquaporin‐4 (AQP4) antibodies in NMO has enhanced researcher attention in this field (Lennon et al., 2004). As a result, numerous papers were published regarding these diseases, and the clinical characteristics and treatment strategies were decided to some degree (Aleixandre‐Benavent et al., 2015; Wingerchuk & Carter, 2014). However, there is increased demand to stratify the current literature regarding idiopathic inflammatory demyelinating disease to serve as a guide to researchers.

Bibliometrics is a research method that analyzes citation frequencies and patterns of articles in a category of interest (Moed, 2009). Although there is some debate as to the association between the number of citations and the quality of the study, highly cited articles can indirectly represent the impact of a particular article on the scientific community and the trends in a specific field of research (Moed, 2009). The results of a citation analysis can suggest “classic lists” of articles in a specific field and “hints” about trends in citations within an area.

Although a few bibliometric studies have examined CIDD, the research objects were restricted to particular regions, time periods or subtypes of CIDD, particularly MS (Aleixandre‐Benavent et al., 2013, 2015; Araujo, Moreira, & Lana‐Peixoto, 2006; Gonzalez de Dios et al., 2013). The aim of this study was to identify and analyze the characteristics of the top 100 most frequently cited articles under the heading of “CIDD” worldwide. Considering the increased importance of NMO in this field and the relatively short time period in which NMO has been regarded as a distinct entity from MS due to its AQP4 antibody specificity, different treatment response and pathology (Wingerchuk et al., 2015), we separately suggested citation classics for NMO in this study.

## 2 Materials and Methods

The Web of Knowledge Journal Citation Reports Science Edition 2014 (Thomson Reuters, New York, NY, USA) was used to search for all journals that are listed under the categories “clinical neurology”, “neuroscience” and “medicine, general & internal”. We retrieved all articles that were cited more than 100 times in the selected journals using the Cited Reference Search option of the Science Citation Index Expanded of the ISI Web of Science database (January 1945–February 2016).

To find the CIDD‐related works among all the articles that had been cited more than 100 times in the three categories, we used following search terms: “multiple sclerosis”, “demyelinating disease”, “myelitis”, “optic neuritis”, “clinically isolated syndrome”, “neuromyelitis optica”, “Devic's disease”, “Balo concentric sclerosis”, “Schilder's diffuse sclerosis”, “Schilder's disease”, “diffuse myelinoclastic sclerosis”, “Marburg multiple sclerosis”, “acute disseminated encephalomyelitis”, “solitary sclerosis”, “acute hemorrhagic leukoencephalitis”, “neuromyelitis optica‐immunoglobulin G (NMO‐IgG)”, and “aquaporin‐4 antibody”. The search terms for the citation classics on NMO included “neuromyelitis optica”, “Devic's disease”, “NMO‐IgG”, and “aquaporin‐4 antibody”. The original texts of all the searched articles were evaluated for their applicability. The citation classics on NMO were defined as articles that were cited more than 100 times (Garfield). The lists of the 100 top cited articles on CIDD and the citation classics on NMO were obtained and analyzed for their characteristics: number of citations, year of publication, published journal, authorship, country and institution of origin, type of article, subject of article (e.g., MS, NMO, ADEM, ON, etc.) and main issues. The country and institution of origin was defined by the affiliation of the first author. If the first author had more than one affiliation, the affiliation of corresponding author was used. Two researchers independently reviewed the data (J.E.K and K.M.P) and any disagreements were decided by further discussion with another neurologist (J.S.B). This study did not need to be reviewed by an ethics committee since it performed a bibliometric analysis of existing published studies.

## 3 Results

### 3.1 Characteristics of the top 100 cited articles of CIDD

**Figure 1:**
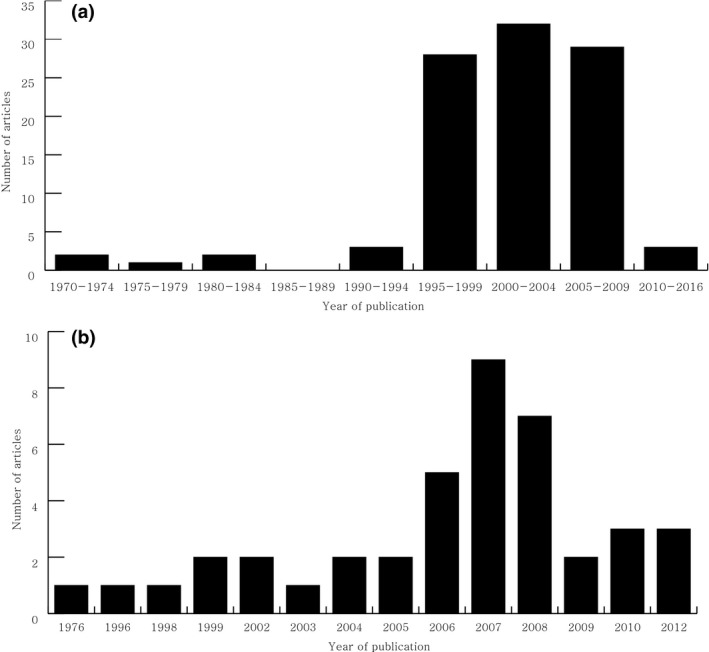
Frequency distribution based on the year of publication for the top 100 cited articles on central nervous system inflammatory demyelinating disease (a) and the citation classics on neuromyelitis optica (b)

The list of the top 100 cited articles on CIDD are presented in Table 1. The average number of citations for the top 100 cited articles were 664 (range, 330–3,897). The articles were published between 1972 and 2011, and the majority of the articles were published after 1995 (Figure 1a). There were 64 articles that were cited more than 400 times, which is the criteria commonly used as the threshold for a citation classic (Garfield). The most frequently cited article was about the recommended diagnostic criteria for MS by an international panel that was published by McDonald et al. in 2001 [rank 1]. The revision of the “McDonald Criteria”, which was published by Polman et al. in 2005 [rank 2], was second highest most cited article (Table 1). Sixty‐four of the 100 articles originated in the United States of America (USA) and the United Kingdom (UK). The Institute of Neurology (London, UK), the Mayo Clinic (Minnesota, USA), the Cleveland Clinic Foundation (Ohio, USA) and the Free University Hospital (Amsterdam, the Netherlands) had the highest rankings for their contribution to the top 100 most cited articles (Table 2). The highly cited articles were published in 13 journals, which was led by *Brain* (*n* = 24) and closely followed by *The New England Journal of Medicine* (*n* = 21) (Table S1). Fifty‐three authors were found to have contributed more than three articles on the list. Miller DH was the most prolific author for the 100 articles, followed by Weinshenker BG and Polman CH (Table S2). Of the top 100 articles, 77 articles were original research articles and 23 articles were review articles or guidelines.

**Table 1:** List of the top 100 cited articles on central nervous system inflammatory demyelinating disease

Rank	Article	No. of citations
1	McDonald WI, Compston A, Edan G et al. Recommended diagnostic criteria for multiple sclerosis: Guidelines from the International Panel on the Diagnosis of Multiple Sclerosis. Ann Neurol 2001;**50**:121–7.	3,897
2	Polman CH, Reingold SC, Edan G et al. Diagnostic criteria for multiple sclerosis: 2005 Revisions to the “McDonald Criteria”. Ann Neurol 2005;**58**:840–6.	2,637
3	Trapp BD, Peterson J, Ransohoff RM, Rudick R, Mörk S, Bö L. Axonal transection in the lesions of multiple sclerosis. N Engl J Med 1998;**338**:278–85.	2,445
4	Lublin FD, Reingold SC. Defining the clinical course of multiple sclerosis: Results of an international survey. Neurology 1996;**46**:907–11.	2,060
5	Noseworthy JH, Lucchinetti C, Rodriguez M et al. Medical progress: Multiple sclerosis. N Engl J Med 2000;**343**:938–52.	1,866
6	Jacobs LD, Cookfair DL, Rudick RA et al. Intramuscular interferon beta‐1 alpha for disease progression in relapsing multiple sclerosis. Ann Neurol 1996;**39**:285–94.	1,726
7	Lucchinetti C, Brück W, Parisi J, Scheithauer B, Rodriquez M, Lassmann H. Heterogeneity of multiple sclerosis lesions: Implications for the pathogenesis of demyelination. Ann Neurol 2000;**47**:707–17.	1,547
8	Polman CH, Reingold SC, Banwell B et al. Diagnostic Criteria for Multiple Sclerosis: 2010 Revisions to the McDonald Criteria. Ann Neurol 2011; **69**:292–302	1,391
9	Wollheim M, Thompson AJ, Waubant E et al. A randomized, placebo‐controlled trial of natalizumab for relapsing multiple sclerosis. N Engl J Med 2006;**354**:899–910.	1,324
10	Wingerchuk DM, Lennon VA, Pittock SJ, Lucchinetti CF, Weinshenker BG. Revised diagnostic criteria for neuromyelitis optica. Neurology 2006;**66**:1485–9.	1,317
11	PRISMS Study Group. Randomised double‐blind placebo‐controlled study of interferon beta‐1a in relapsing/remitting multiple sclerosis. Lancet 1998;**352**:1498–504.	1,310
12	Lennon VA, Wingerchuk DM, Kryzer TJ et al. A serum autoantibody marker of neuromyelitis optica: distinction from multiple sclerosis. Lancet 2004;**364**:2106–12.	1,231
13	Compston A, Coles A. Multiple sclerosis. Lancet 2008;**372**:1502–17.	1,151
14	Ferguson B, Matyszak MK, Esiri MM, Perry VH. Axonal damage in acute multiple sclerosis lesions. Brain 1997;**120**:393–9.	976
15	Jacobs LD, Beck RW, Simon JH et al. Intramuscular interferon beta‐1a therapy initiated during a first demyelinating event in multiple sclerosis. Am J Opthalmol 2001;**131**:154–5.	946
16	The International Multiple Sclerosis Genetics Consortium. Risk alleles for multiple sclerosis identified by a genomewide study. N Engl J Med 2007;**357**:851–62.	913
17	Wingerchuk DM, Hogancamp WF, O'Brien PC, Weinshenker BG. The clinical course of neuromyelitis optica (Devic's syndrome). Neurology 1999;**53**:1107–14.	886
18	Hauser SL, Waubant E, Arnold DL, Vollmer T et al. B‐cell depletion with Rituximab in relapsing‐remitting multiple sclerosis. N Engl J Med 2008;**358**:676–88.	830
19	Kappos L, Radue EW, O'Connor P et al. A Placebo‐Controlled Trial of Oral Fingolimod in Relapsing Multiple Sclerosis. N Engl J Med 2010;**362**:387–401.	819
20	Munger KL, Levin LI, Hollis BW et al. Serum 25‐hydroxyvitamin D levels and risk of multiple sclerosis. JAMA 2006;**296**:2832–8.	771
21	Barkhof F, Filippi M, Miller DH et al. Comparison of MRI criteria at first presentation to predict conversion to clinically definite multiple sclerosis. Brain 1997;**120**:2059–69.	767
22	Wingerchuk DM, Lennon VA, Lucchinetti CF, Pittock SJ, Weinshenker BG. The spectrum of neuromyelitis optica. Lancet Neurol 2007;**6**:805–15.	757
23	Cohen JA, Barkhof F, Comi G et al. Oral Fingolimod or Intramuscular Interferon for Relapsing Multiple Sclerosis. N Engl J Med 2010;**362**:402–15.	744
24	Frohman EM, Racke MK, Raine CS. Medical progress: Multiple sclerosis—The plaque and its pathogenesis. N Engl J Med 2006; **354**:942–55.	742
25	Miller DH, Khan OA, Sheremata WA et al. A controlled trial of natalizumab for relapsing multiple sclerosis. N Engl J Med 2003;**348**:15–23.	741
26	Peterson JW, Bö L, Mörk S, Chang A, Trapp BD. Transected neurites, apoptotic neurons, and reduced inflammation in cortical multiple sclerosis lesions. Ann Neurol 2001;**50**:389–400.	733
27	Kutzelnigg A, Lucchinetti CF, Stadelmann C et al. Cortical demyelination and diffuse white matter injury in multiple sclerosis. Brain. 2005;**128**:2705–12.	683
28a	Confavreux C, Hutchinson M, Hours MM, Cortinovis‐Tourniaire P, Moreau T. Rate of pregnancy‐related relapse in multiple sclerosis. N Engl J Med 1998;**339**:285–91.	663
28b	European Study Group on Interferon beta‐1b in Secondary progressive MS. Placebo‐controlled multicentre randomised trial of interferon beta‐1b in treatment of secondary progressive multiple sclerosis. Lancet 1998;**352**:1491–7.	663
30	Comi G, Filippi M, Barkhof F et al. Effect of early interferon treatment on conversion to definite multiple sclerosis: a randomised study. Lancet 2001;**357**:1576–82.	631
31	Confavreux C, Vukusic S, Moreau T et al. Relapses and progression of disability in multiple sclerosis. N Engl J Med 2000;**343**:1430–8.	619
32	Kleinschmidt‐DeMasters BK, Tyler KL. Progressive multifocal leukoencephalopathy complicating treatment with natalizumab and interferon beta‐1a for multiple sclerosis. N Engl J Med 2005;**353**:369–74.	617
33	Rudick RA, Stuart WH, Calabresi PA et al. Natalizumab plus interferon beta‐1a for relapsing multiple sclerosis. N Engl J Med 2006; **354**: 911–23.	611
34	Lucchinetti CF, Mandler RN, McGavern D et al. A role for humoral mechanisms in the pathogenesis of Devic's neuromyelitis optica. Brain 2002;**125**:1450–61.	587
35	Kappos L, Antel J, Comi G et al. Oral fingolimod (FTY720) for relapsing multiple sclerosis. N Engl J Med 2006;**355**:1124–40.	585
36	Beck RW, Cleary PA, Anderson MM et al. A randomized, controlled trial of corticosteroids in the treatment of acute optic neuritis. N Engl J Med 1992;**326**:581–8.	582
37	Cutter GR, Baier ML, Rudick RA et al. Development of a multiple sclerosis functional composite as a clinical trial outcome measure. Brain 1999;**122**:871–82.	539
38	Bitsch A, Schuchardt J, Bunkowski S, Kuhlmann T, Brück W. Acute axonal injury in multiple sclerosis—Correlation with demyelination and inflammation. Brain 2000;**123**:1174–83.	536
39	Trapp BD, Nave KA. Multiple sclerosis: An immune or neurodegenerative disorder? Annu Rev Neurosci 2008;**31**:247–69.	531
40	Goodin DS, Frohman EM, Garmany GP Jr et al. Disease modifying therapies in multiple sclerosis—Report of the Therapeutics and Technology Assessment Subcommittee of the American Academy of Neurology and the MS Council for Clinical Practice Guidelines. Neurology 2002;**58**:169–78.	518
41	Halliday AM, McDonald WI, Mushin J. Delayed visual evoked response in optic neuritis. Lancet 1972;**1**:982–5.	509
42	Munger KL, Zhang SM, O'Reilly E et al. Vitamin D intake and incidence of multiple sclerosis. Neurology 2004;**62**:60–5.	503
43	Barnett MH, Prineas JW. Relapsing and remitting multiple sclerosis: Pathology of the newly forming lesion. Ann Neurol 2004;**55**:458–68.	496
44	Gold R, Linington C, Lassmann H. Understanding pathogenesis and therapy of multiple sclerosis via animal models: 70 years of merits and culprits in experimental autoimmune encephalomyelitis research. Brain 2006;**129**:1953–71.	491
45	Brex PA, Ciccarelli O, O'Riordan JI, Sailer M, Thompson AJ, Miller DH. A longitudinal study of abnormalities on MRI and disability from multiple sclerosis. N Engl J Med 2002;**346**:158–64.	480
46	Hartung HP, Gonsette R, König N et al. Mitoxantrone in progressive multiple sclerosis: a placebo‐controlled, double‐blind, randomised, multicentre trial. Lancet 2002;**360**:2018–25.	474
47	Chang A, Tourtellotte WW, Rudick R, Trapp BD. Premyelinating oligodendrocytes in chronic lesions of multiple sclerosis. N Engl J Med 2002;**346**:165–73.	473
48	Comi G, Filippi M, Wolinsky JS. European/Canadian multicenter, double‐blind, randomized, placebo‐controlled study of the effects of glatiramer acetate on magnetic resonance imaging‐measured disease activity and burden in patients with relapsing multiple sclerosis. Ann Neurol 2001;**49**:290–7.	466
49	Kidd D, Barkhof F, McConnell R, Algra PR, Allen IV, Revesz T. Cortical lesions in multiple sclerosis. Brain 1999;**122**:17–26.	461
50	Young IR, Hall AS, Pallis CA, Legg NJ, Bydder GM, Steiner RE.Nuclear magnetic resonance imaging of the brain in multiple sclerosis. Lancet 1981;**2**:1063–6.	459
51	Van Waesberghe JH, Kamphorst W, De Groot CJ et al. Axonal loss in multiple sclerosis lesions: Magnetic resonance imaging insights into substrates of disability. Ann Neurol 1999;**46**:747–54.	457
52	Franklin RJ. Why does remyelination fail in multiple sclerosis? Nat Rev Neurosci 2002;**3**:705–14.	439
53	Losseff NA, Webb SL, O'Riordan JI et al. Spinal cord atrophy and disability in multiple sclerosis—A new reproducible and sensitive MRI method with potential to monitor disease progression. Brain 1996;**119**:701–18.	436
54	Jersild C, Fog T, Hansen GS, Thomsen M, Svejgaard A, Dupont B. Histocompatibility determinants in multiple sclerosis, with special reference to clinical course. Lancet 1973;**2**:1221–5.	430
55	The CAMMS223 Trial Investigators. Alemtuzumab vs. Interferon beta‐1a in early multiple sclerosis. N Engl J Med 2008;**359**:1786–1801.	428
56	van Oosten BW, Barkhof F, Truyen L et al. Increased MRI activity and immune activation in two multiple sclerosis patients treated with the monoclonal anti‐tumor necrosis factor antibody cA2. Neurology 1996;**47**:1531–4.	422
57	Confavreux C, Vukusic S, Adeleine P. Early clinical predictors and progression of irreversible disability in multiple sclerosis: an amnesic process. Brain 2003;**126**:770–82.	419
58a	Ascherio A, Munger KL. Environmental risk factors for multiple sclerosis. Part I: The role of infection. Ann Neurol 2007;**61**:288–99.	417
58b	van Walderveen MA, Kamphorst W, Scheltens P et al. Histopathologic correlate of hypointense lesions on T1‐weighted spin‐echo MRI in multiple sclerosis. Neurology 1998;**50**:1282–8.	417
60	Kuhlmann T, Lingfeld G, Bitsch A, Schuchardt J, Brück W. Acute axonal damage in multiple sclerosis is most extensive in early disease stages and decreases over time. Brain 2002;125:2202–12.	415
61	Werring DJ, Clark CA, Barker GJ, Thompson AJ, Miller DH. Diffusion tensor imaging of lesions and normal‐appearing white matter in multiple sclerosis. Neurology 1999;**52**:1626–32.	410
62	Confavreux C, Aimard G, Devic M. Course and prognosis of multiple sclerosis assessed by the computerized data processing of 349 patients. Brain 1980;**103**:281–300.	406
63	Chang A, Nishiyama A, Peterson J, Prineas J, Trapp BD. NG2‐positive oligodendrocyte progenitor cells in adult human brain and multiple sclerosis lesions. J Neurosci 2000;**20**:6404–12.	401
64	Beck RW, Cleary PA, Trobe JD et al. The effect of corticosteroids for acute optic neuritis on the subsequent development of multiple sclerosis. N Engl J Med 1993;**329**:1764–9.	400
65	Miller DH, Grossman RI, Reingold SC, McFarland HF. The role of magnetic resonance techniques in understanding and managing multiple sclerosis. Brain 1998;**121**:3–24.	395
66	Miller DH, Albert PS, Barkhof F et al. Guidelines for the use of magnetic resonance techniques in monitoring the treatment of multiple sclerosis. Ann Neurol 1996;**39**:6–16.	394
67	Chiaravalloti ND, DeLuca J. Cognitive impairment in multiple sclerosis. Lancet Neurol 2008;**7**:1139–51.	391
68	Weinshenker BG, O'Brien PC, Petterson TM et al. A randomized trial of plasma exchange in acute central nervous system inflammatory demyelinating disease. Ann Neurol 1999;**46**:878–86.	380
69	Rose AS, Ellison GW, Myers LW, Tourtellotte WW. Criteria for the clinical diagnosis of multiple sclerosis. Neurology 1976;**26**:20–2.	377
70	Kappos L, Polman CH, Freedman MS et al. Treatment with interferon beta‐1b delays conversion to clinically definite and McDonald MS in patients with clinically isolated syndromes. Neurology 2006;**67**:1242–9.	375
71	Krupp LB, Banwell B, Tenembaum S. Consensus definitions proposed for pediatric multiple sclerosis and related disorders. Neurology 2007;**68**:S7–12.	373
72a	De Stefano N, Matthews PM, Fu L et al. Axonal damage correlates with disability in patients with relapsing‐remitting multiple sclerosis—Results of a longitudinal magnetic resonance spectroscopy study. Brain 1998;**121**:1469–77.	370
72b	Pittock SJ, Lennon VA, Krecke K, Wingerchuk DM, Lucchinetti CF, Weinshenker BG. Brain abnormalities in neuromyelitis optica. Arch Neurol 2006;**63**:390–6.	370
74	Losseff NA, Wang L, Lai HM et al. Progressive cerebral atrophy in multiple sclerosis—A serial MRI study. Brain 1996;**119**:2009–19.	369
75	Petajan JH, Gappmaier E, White AT, Spencer MK, Mino L, Hicks RW. Impact of aerobic training on fitness and quality of life in multiple sclerosis. Ann Neurol 1996;**39**:432–41.	367
76	Zamboni P, Galeotti R, Menegatti E et al. Chronic cerebrospinal venous insufficiency in patients with multiple sclerosis. J Neurol Neurosurg Psychiatry 2009;**80**:392–9.	365
77	Roemer SF, Parisi JE, Lennon VA et al. Pattern‐specific loss of aquaporin‐4 immunoreactivity distinguishes neuromyelitis optica from multiple sclerosis. Brain 2007;**130**:1194–205.	363
78	Magliozzi R, Howell O, Vora A et al. Meningeal B‐cell follicles in secondary progressive multiple sclerosis associate with early onset of disease and severe cortical pathology. Brain 2007;**130**:1089–104.	356
79	Truyen L, van Waesberghe JH, van Walderveen MA et al. Accumulation of hypointense lesions (“black holes”) on T‐1 spin‐echo MRI correlates with disease progression in multiple sclerosis. Neurology 1996;**47**:1469–76.	355
80	Fu L, Matthews PM, De Stefano N et al. Imaging axonal damage of normal‐appearing white matter in multiple sclerosis. Brain 1998;**121**:103–13.	354
81a	Dale RC, de Sousa C, Chong WK, Cox TC, Harding B, Neville BG. Acute disseminated encephalomyelitis, multiphasic disseminated encephalomyelitis and multiple sclerosis in children. Brain 2000;**123**;2407–22.	352
81b	Roxburgh RH, Seaman SR, Masterman T et al. Multiple sclerosis severity score—Using disability and disease duration to rate disease severity. Neurology 2005;**64**:1144–51.	352
83	Dutta R, McDonough J, Yin X et al. Mitochondrial dysfunction as a cause of axonal degeneration in multiple sclerosis patients. Ann Neurol 2006;59:478–89.	351
84	Banati RB, Newcombe J, Gunn RN et al. The peripheral benzodiazepine binding site in the brain in multiple sclerosis—Quantitative in vivo imaging of microglia as a measure of disease activity. Brain 2000;**123**:2321–37.	350
85a	Hemmer B, Archelos JJ, Hartung HP. New concepts in the immunopathogenesis of multiple sclerosis. Nat Rev Neurosci 2002;**3**:291–301.	346
85b	Zajicek J, Fox P, Sanders H et al. Cannabinoids for treatment of spasticity and other symptoms related to multiple sclerosis (CAMS study): multicentre randomised placebo‐controlled trial. Lancet 2003;**362**:1517–26.	346
87	Coles AJ, Wing MG, Molyneux P et al. Monoclonal antibody treatment exposes three mechanisms underlying the clinical course of multiple sclerosis. Ann Neurol 1999;**46**:296–304.	344
88a	Durelli L, Verdun E, Barbero P et al. Every‐other‐day interferon beta‐1b versus once‐weekly interferon beta‐1a for multiple sclerosis: results of a 2‐year prospective randomised multicentre study (INCOMIN). Lancet 2002;**359**:1453–60.	343
88b	Kesselring J, Miller DH, Robb SA et al. Acute disseminated encephalomyelitis – MRI findings and the distinction from multiple sclerosis. Brain 1990;**113**:291–302.	343
90a	Cree BA, Lamb S, Morgan K, Chen A, Waubant E, Genain C. An open label study of the effects of rituximab in neuromyelitis optica. Neurology 2005;**64**:1270–2.	342
90b	Miller DH, Barkhof F, Frank JA, Parker GJ, Thompson AJ. Measurement of atrophy in multiple sclerosis: pathological basis, methodological aspects and clinical relevance. Brain 2002;**125**:1676–95.	342
92	Berger T, Rubner P, Schautzer F et al. Antimyelin antibodies as a predictor of clinically definite multiple sclerosis after a first demyelinating event. N Engl J Med 2003;**349**:139–45.	341
93a	Johnson KP, Brooks BR, Cohen JA et al. Extended use of glatiramer acetate (Copaxone) is well tolerated and maintains its clinical effect on multiple sclerosis relapse rate and degree of disability. Neurology 1998;**50**:701–8.	340
93b	Vollmer T, Key L, Durkalski V et al. Oral simvastatin treatment in relapsing‐remitting multiple sclerosis. Lancet 2004;**363**:1607–8.	340
95	Pittock SJ, Weinshenker BG, Lucchinetti CF, Wingerchuk DM, Corboy JR, Lennon VA. Neuromyelitis optica brain lesions localized at sites of high aquaporin 4 expression. Arch Neurol 2006;**63**:964–8.	338
96	Wolswijk G. Chronic stage multiple sclerosis lesions contain a relatively quiescent population of oligodendrocyte precursor cells. J Neurosci 1998;**18**:601–9.	337
97	Frischer JM, Bramow S, Dal‐Bianco A et al. The relation between inflammation and neurodegeneration in multiple sclerosis brains. Brain 2009;**132**:1175–89.	336
98	O'Riordan JI, Thompson AJ, Kingsley DP et al. The prognostic value of brain MRI in clinically isolated syndromes of the CNS—A 10‐year follow‐up. Brain 1998;121:495–503.	334
99	Minagar A, Shapshak P, Fujimura R, Ownby R, Heyes M, Eisdorfer C. The role of macrophage/microglia and astrocytes in the pathogenesis of three neurologic disorders: HIV‐associated dementia, Alzheimer disease, and multiple sclerosis. J Neurol Sci 2002;**202**:13–23.	331
100	Filippi M, Cercignani M, Inglese M, Horsfield MA, Comi G. Diffusion tensor magnetic resonance imaging in multiple sclerosis. Neurology 2001;**56**:304–11.	330

**Table 2:** Countries and institutions of origin for the citation classics in the field of central nervous system inflammatory demyelinating disease (a) and neuromyelitis optica (b)

	No. of Citation Classics
(a) CNS inflammatory demyelinating disease	
Countries of origin	
USA	41
UK	23
The Netherlands	9
Italy	6
Germany	5
France	4
Switzerland	4
Canada	3
Austria	3
Australia	1
Denmark	1
Institutions of origin	
Institute of Neurology (London, UK)	12
Mayo Clinic, Rochester (Minnesota, USA)	11
Cleveland Clinic Foundation (Ohio, USA)	8
Free University Hospital (Amsterdam, Netherlands)	8
University of Cambridge (Cambridge, UK)	5
Harvard Medical School (Boston, USA)	4
Hôpital Neurologique (Lyon, France)	4
University Hospital Basel (Basel, Switzerland)	4
Scientific Institute Ospedale San Raffaele, University of Milan (Milan, Italy)	3
University of California (San Francisco, USA)	3
Center for Brain Research, Medical University of Vienna (Vienna, Austria)	2
Montreal Neurological Institute and Hospital (Quebec, Canada)	2
State University of New York School of Medicine, Buffalo and Buffalo General Hospital (New York, USA)	2
University of South Florida College of Medicine (Florida, USA)	2
(b) Neuromyelitis optica	
Countries of origin	
USA	20
UK	6
Japan	5
Germany	4
Austria	2
Italy	2
Canada	1
France	1
Institutions of origin	
Mayo Clinic, Rochester (Minnesota, USA)	16
Tohoku University (Sendai, Japan)	4
University of Oxford (Oxford, UK)	3
St George's, University of London (London, UK)	2
University of Heidelberg (Heidelberg, Germany)	2

CNS, central nervous system; UK, United Kingdom; USA, United States of America.

The most predominant subject of the 100 articles was MS (*n* = 85) and among these, 7 articles focused on evaluating prognostic factors (clinical, magnetic resonance imaging [MRI] features or treatment availability) to determine the conversion of clinically isolated syndrome to MS [ranks 21,30,45,64,70,92,98 in Table 1]. Two articles enrolled ADEM patients, which were mainly pediatrics, and discussed the clinical and MRI features of ADEM that can be used to differentiate ADEM from MS [ranks 81a,88b in Table 1]. Two articles enrolled patients with several subtypes of CIDD concomitantly and one of these articles was a randomized controlled trial that evaluated the effect of plasma exchange in acute central nervous system (CNS) inflammatory disease [ranks 68,71 in Table 1]. Only nine articles were about NMO [ranks 10,12,17,22,34,72b,77,90a,95 in Table 1], and two articles about ON [ranks 36,41 in Table 1] were also included in the list.

**Figure 2:**
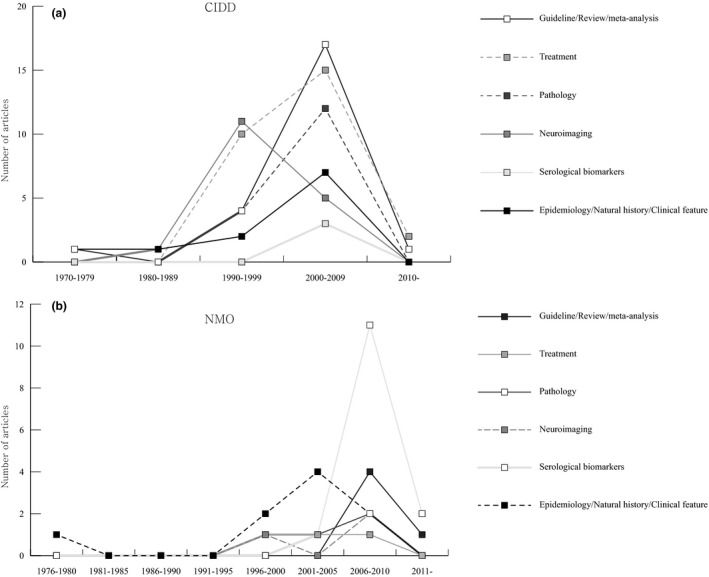
Time trends in categories of citation classics on central nervous system inflammatory demyelinating disease (a) and neuromyelitis optica (b). CIDD, Central nervous system inflammatory demyelinating disease; NMO, Neuromyelitis optica

The main issues and their time trends of the highly cited articles are summarized in Table 3 and Figure 2a. Among the original articles, the majority of the papers (*n* = 27) focused on treatment topics such as the effects of immunomodulatory drugs including intramuscular interferon β‐1a, glatiramer acetate, natalizumab, rituximab, fingolimod, mitoxantrone, alemtuzumab, monoclonal anti‐tumor necrosis factor antibody cA2, steroid, and plasma exchange [ranks 6,9,11,15,18,19,23,25,28b,30,33,35,36,46,48,55,56,64,68,70,87,88a,90a,93a,93b in Table 1]. Other common issues were neuroimaging features as evaluated by MRI, diffusion tensor imaging, magnetic resonance spectroscopy and positron emission tomography (*n* = 17). A considerable portion of the papers also discussed the pathological findings of CIDD (*n* = 16).

**Table 3:** General issues discussed in the highly cited articles on central nervous system inflammatory demyelinating disease and neuromyelitis optica

Category	No. of articles
CNS inflammatory demyelinating disease	
Epidemiology and natural history	11
Clinical scoring	1
Diagnostic/prognostic biomarkers	
Serological biomarkers	3
Neuroimaging	17
Electrophysiology (visual evoked potential)	1
Pathology	16
Treatment	27
Treatment‐related complications	1
Guideline/review/meta‐analysis	23
Neuromyelitis optica	
Epidemiology/natural history/clinical feature	9
Diagnostic/prognostic biomarkers	
Serological biomarkers	14
Neuroimaging	3
Pathology	3
Treatment	3
Guideline/review/meta‐analysis	5
Basic research	4

CNS, central nervous system.

### 3.2 Citation classics of NMO

Using the cut‐off threshold of 100 citations, we found 41 citation classics for NMO (Table 4). The mean number of citations of the citation classics was 291 (range, 102–1,317). All articles were published between 1976 and 2012 and there was a surge in the number of publications after 2006 (Figure 1b). This surge in publications corresponds to the publication of the top 2 cited articles, in which the NMO‐specific autoantibody (NMO‐IgG) was detected in the serum of NMO patients [rank 2 in Table 4] and was subsequently followed by the suggestion of revised diagnostic criteria that included NMO‐IgG positivity in the diagnosis [rank 1 in Table 4]. These two articles had a significant role in discriminating NMO from MS. There are eight different countries of origin for the citation classics (Table 2). USA (*n* = 20) had the largest number of articles, which was followed by the UK (*n* = 6), Japan (*n* = 5) and Germany (*n* = 4). The Mayo Clinic (Minnesota, USA) was the most active publishing institution for the NMO citation classics (Table 2). The citation classics were published in 13 journals, which was led by *Neurology* (*n* = 15), followed by *Brain* (*n* = 7), *Archives of Neurology* (*n* = 5) and *Annals of Neurology* (*n* = 3; Table S1). Twenty‐four authors contributed 3 or more of the articles on the citation classics list. Weinshenker BG had the highest number of articles, followed by Wingerchuk DM, Pittock SJ, Lennon VA, and Lucchinetti CF (Table S3). Among the 41 articles, 5 were review articles or guidelines [ranks 1,4,28,29,40 in Table 4], 1 was a case report [rank 37 in Table 4], and the remainder of the articles was original research articles. Four articles reported basic science studies [ranks 14,16,17,20 in Table 4]. The main issues discussed in the citation classics for NMO and their trends over time are summarized in Table 3 and Figure 2b. The most frequently discussed topic among the citation classics was the serological markers, NMO‐IgG or AQP4 antibody. The original studies concerning the diagnostic and prognostic values of NMO‐IgG or the AQP4 antibody and their pathogenicity were frequently cited [ranks 2,10,11,14,15,16,17,20,21,22,23,24,31,33,34,35,36,41 in Table 4]. The second most commonly cited issues were related to the natural course and clinical features of NMO [ranks 3,12,25,26,27,30,32,37,39 in Table 4]. Other frequently cited issues included NMO pathology [ranks 5,7,13 in Table 4], the effect of various treatments (rituximab or prednisolone with azathioprine) [ranks 8,18,19 in Table 4], and MRI characteristics [ranks 6,9,38 in Table 4].

**Table 4:** Citation classics on neuromyelitis optica

Rank	Article	No. of citations
1	Wingerchuk DM, Lennon VA, Pittock SJ, Lucchinetti CF, Weinshenker BG. Revised diagnostic criteria for neuromyelitis optica. Neurology 2006;**66**:1485–9.	1,317
2	Lennon VA, Wingerchuk DM, Kryzer TJ et al. A serum autoantibody marker of neuromyelitis optica: distinction from multiple sclerosis. Lancet 2004;**364**:2106–12.	1,231
3	Wingerchuk DM, Hogancamp WF, O'Brien PC, Weinshenker BG. The clinical course of neuromyelitis optica (Devic's syndrome). Neurology 1999;**53**:1107–14.	886
4	Wingerchuk DM, Lennon VA, Lucchinetti CF, Pittock SJ, Weinshenker BG. The spectrum of neuromyelitis optica. Lancet Neurol 2007;**6**:805–15.	757
5	Lucchinetti CF, Mandler RN, McGavern D et al. A role for humoral mechanisms in the pathogenesis of Devic's neuromyelitis optica. Brain. 2002;**125**:1450–61.	587
6	Pittock SJ, Lennon VA, Krecke K, Wingerchuk DM, Lucchinetti CF, Weinshenker BG. Brain abnormalities in neuromyelitis optica. Arch Neurol 2006;**63**:390–6.	370
7	Roemer SF, Parisi JE, Lennon VA et al. Pattern‐specific loss of aquaporin‐4 immunoreactivity distinguishes neuromyelitis optica from multiple sclerosis. Brain 2007;**130**:1194–205.	363
8	Cree BA, Lamb S, Morgan K, Chen A, Waubant E, Genain C. An open label study of the effects of rituximab in neuromyelitis optica. Neurology 2005;**64**;1270–2.	342
9	Pittock SJ, Weinshenker BG, Lucchinetti CF, Wingerchuk DM, Corboy JR, Lennon VA. Neuromyelitis optica brain lesions localized at sites of high aquaporin 4 expression. Arch Neurol 2006;**63**:964–8.	338
10	Takahashi T, Fujihara K, Nakashima I et al. Anti‐aquaporin‐4 antibody is involved in the pathogenesis of NMO: a study on antibody titre. Brain 2007;**130**:1235–43.	319
11	Weinshenker BG, Wingerchuk DM, Vukusic S et al. Neuromyelitis optica IgG predicts relapse after longitudinally extensive transverse myelitis. Ann Neurol 2006;**59**:566–9.	307
12	O'Riordan JI, Gallagher HL, Thompson AJ et al. Clinical, CSF, and MRI findings in Devic's neuromyelitis optica. J Neurol Neurosurg Psychiatry. 1996;**60**:382–7.	276
13	Misu T, Fujihara K, Kakita A et al. Loss of aquaporin 4 in lesions of neuromyelitis optica: distinction from multiple sclerosis. Brain 2007;**130**:1224–34.	276
14	Bradl M, Misu T, Takahashi T et al. Neuromyelitis optica: pathogenicity of patient immunoglobulin in vivo. Ann Neurol 2009;**66**:630–43.	266
15	Pittock SJ, Lennon VA, de Seze J et al. Neuromyelitis optica and non‐organ‐specific Autoimmunity. Arch Neurol 2008;**65**:78–83.	240
16	Bennett JL, Lam C, Kalluri SR et al. Intrathecal pathogenic anti‐aquaporin‐4 antibodies in early neuromyelitis optica. Ann Neurol 2009;66:617–29.	221
17	Hinson SR, Pittock SJ, Lucchinetti CF et al. Pathogenic potential of IgG binding to water channel extracellular domain in neuromyelitis optica. Neurology 2007;**69**:2221–31.	219
18	Jacob A, Weinshenker BG, Violich I et al. Treatment of neuromyelitis optica with rituximab retrospective analysis of 25 Patients. Arch Neurol 2008;65:1443–8.	212
19	Mandler RN, Ahmed W, Dencoff JE. Devic's neuromyelitis optica: A prospective study of seven patients treated with prednisone and azathioprine. Neurology 1998;**51**:1219–20.	210
20	Saadoun S, Waters P, Bell BA et al. Intra‐cerebral injection of neuromyelitis optica immunoglobulin G and human complement produces neuromyelitis optica lesions in mice. Brain 2010;**133**:349–61.	210
21	Jarius S, Aboul‐Enein F, Waters P, Vincent A, Verkman AS, Papadopoulos MC. Antibody to aquaporin‐4 in the long‐term course of neuromyelitis optica. Brain 2008;**131**:3072–80.	199
22	Matsuoka T, Matsushita T, Kawano Y et al. Heterogeneity of aquaporin‐4 autoimmunity and spinal cord lesions in multiple sclerosis in Japanese. Brain 2007;**130**:1206–23.	181
23	Matiello M, Lennon VA, Jacob A et al. NMO‐IgG predicts the outcome of recurrent optic neuritis. Neurology 2008;**70**:2197–200.	180
24	Waters P, Jarius S, Littleton E et al. Aquaporin‐4 antibodies in neuromyelitis Optica and longitudinally extensive transverse myelitis. Arch Neurol 2008;**65**:913–9.	176
25	Wingerchuk DM, Weinshenker BG. Neuromyelitis optica—Clinical predictors of a relapsing course and survival. Neurology 2003;**60**:848–53.	173
26	Nakashima I, Fujihara K, Miyazawa I et al. Clinical and MRI features of Japanese patients with multiple sclerosis positive for NMO‐IgG. J Neurol Neurosurg Psychiatry 2006;**77**:1073–5.	165
27	Ghezzi A, Bergamaschi R, Martinelli V et al. Clinical characteristics, course and prognosis of relapsing Devic's Neuromyelitis Optica. J Neurol 2004;**251**:47–52.	163
28	Jarius S, Wildemann B. AQP4 antibodies in neuromyelitis optica: diagnostic and pathogenetic relevance. Nat Rev Neurol 2010;**6**:383–92.	160
29	Sellner J, Boggild M, Clanet M et al. EFNS guidelines on diagnosis and management of neuromyelitis optica. Eur J Neurol 2010;**17**:1019–32.	144
30	Misu T, Fujihara K, Nakashima I, Sato S, Itoyama Y. Intractable hiccup and nausea with periaqueductal lesions in neuromyelitis optica. Neurology 2005;**65**:1479–82.	143
31	Paul F, Jarius S, Aktas O et al. Antibody to aquaporin 4 in the diagnosis of neuromyelitis optica. PLos Med 2007;**4**:e133.	132
32	de Seze J, Stojkovic T, Ferriby D et al. Devic's neuromyelitis optica: clinical, laboratory, MRI and outcome profile. J Neurol Sci 2002;**197**:57–61.	130
33	McKeon A, Lennon VA, Lotze T et al. CNS aquaporin‐4 autoimmunity in children. Neurology. 2008;**71**:93–100.	130
34	Waters PJ, McKeon A, Leite MI et al. Serologic diagnosis of NMO A multicenter comparison of aquaporin‐4‐IgG assays. Neurology 2012;**78**:665–71.	128
35	Jarius S, Franciotta D, Bergamaschi R et al. NMO‐IgG in the diagnosis of neuromyelitis optica. Neurology 2007:**68**:1076–7.	127
36	Banwell B, Tenembaum S, Lennon VA et al. Neuromyelitis optica‐IgG in childhood inflammatory demyelinating CNS disorders. Neurology 2008;**70**:344–52.	110
37	April RS, Vansonnenberg E. A case of neuromyelitis optica (Devic's syndrome) in systemic lupus erythematosus. Clinicopathologic report and review of the literature. Neurology 1976;**26**:1066–70.	109
38	Filippi M, Rocca MA, Moiola L et al. MRI and magnetization transfer imaging changes in the brain and cervical cord of patients with Devic's neuromyelitis optica. Neurology 1999;**53**:1705–10.	108
39	Wingerchuk DM, Pittock SJ, Lucchinetti CF, Lennon VA, Weinshenker BG. A secondary progressive clinical course is uncommon in neuromyelitis optica. Neurology 2007;**68**:603–5.	105
40	Papadopoulos MC, Verkman AS. Aquaporin 4 and neuromyelitis optica. Lancet Neurol 2012;**11**:535–44.	103
41	Jarius S, Ruprecht K, Wildemann B et al. Contrasting disease patterns in seropositive and seronegative neuromyelitis optica: A multicentre study of 175 patients. J Neuroinflammation 2012;**9**:14.	102

## 4 Discussion

In the current study, we identified and characterized the top 100 cited articles concerning CIDD. Using this bibliometric approach, we can examine the historical progress within a field of interest and inform researchers of the articles or authors that have had a significant impact on the field.

The majority of the top 100 frequently cited articles on CIDD were published during 1995–2009. This unequal distribution might reflect the natural life span of articles in a specific academic society. Typically, scientific articles begin to be cited 1 to 2 years after publication and reach a maximum between 3 and 10 years (Marx, Schier, & Wanitschek, 2001). After this period, the number of citations for a particular article gradually decrease due to the “obliteration by incorporation” phenomenon, where the results of a citation classic are absorbed into the current knowledge (Garfield, 1987). Therefore, similar to the findings from other studies, a citation analysis can accurately capture the true impact of articles published within the last 10–20 years. Only 3 of the top 100 articles were published in 2010–2016. This result does not suggest that the recently published articles were less important, but indicates that insufficient time has elapsed to accumulate a large number of citations. Therefore, it is important to not only examine the absolute number of citations of an article but also the entire list of published articles to avoid overlooking critical works in that field. For example, the article describing the diagnostic criteria for MS that was published in 1976, which ranked 69 in our list, did not have less influence on researchers studying MS than the study on the McDonald criteria, which was published later and had the top rank in our study. The distribution of publication years of the NMO citation classics showed a somewhat different pattern. The number of citation classics abruptly increased after 2006. Before the detection of NMO‐specific antibodies (NMO‐IgG and AQP4‐antibody), NMO was regarded as a rare form of MS. “Optico‐spinal MS” was historical used to describe this unique phenotype (Kuroiwa & Shibasaki, 1976). However, the publication of revised NMO diagnostic criteria in 2006 that included MRI diagnostic criteria according to the length of the spinal cord lesion and the detection of the AQP4 antibody led many researchers to distinguish NMO from MS (Wingerchuk, Lennon, Pittock, Lucchinetti, & Weinshenker, 2006), which increased the importance of investigating NMO specifically. This conceptual change in NMO diagnosis for several decades might be indirectly reflected in our study.

About two thirds of articles in both lists (CIDD and NMO) originated from USA and United Kingdom. The overwhelming influence of these two countries can also be found in other clinical disciplines. These findings might be explained by the larger community of specialists, the availability of research funds and organized support in this area and the tendency of American/British researchers to cite local papers. In addition to these two countries, the Netherlands, Italy and Germany produced a relatively large number of highly cited articles on CIDD. Japan was the only non‐western country that had a significant number of NMO citation classics, which might reflect the ethnic predilection of NMO in Asia and the research efforts of the Institute of Tohoku University (Kira, 2003).

Journal impact factor is considered a representative metric for the influence of that journal in the associated scientific field (Garfield, 2006). Eighty‐eight percent of the top 100 cited articles on CIDD were published in five major journals with high impact factors: *Brain, The New England Journal of Medicine, Neurology, Annals of Neurology,* and *The Lancet*. However, not all of the frequently cited articles were published in high impact factor journals, but were published in journals with rich histories. The lead high impact factor journals were the same for the NMO citation classics list as for the CIDD list. However, the number of the journals in the Web of Science category “medicine, general & internal” was higher in the CIDD list than the NMO list.

Six of the articles among top 10 most cited articles on CIDD were about proposed diagnostic criteria guidelines for MS or NMO or review articles concerning the pathogenesis or clinical definition of these two diseases. The significant influence of these guidelines to individual clinical or experimental studies is expected considering their essential use in clinical practice and research. Common issues among the CIDD articles focused on the results from randomized controlled trials on the use of immunomodulatory drugs, the pathogenesis of MS and the diagnostic and prognostic use of new neuroimaging technologies such as MRI. The most common issues among the NMO articles were related to the pathogenicity of NMO and the diagnostic/prognostic value of the NMO‐IgG or AQP4 antibodies. Articles discussing the treatment and neuroimaging issues surrounding NMO represented only a small portion of the current list, but is likely to grow fast based on the trends observed in MS over the last few decades.

The number of citation classics, which was defined as articles that received more than 400 citations (Garfield), was 64 for the CIDD list. This number is slightly smaller than other neurological diseases (107 for Parkinson's disease and 89 for epilepsy; Ibrahim, Snead, Rutka, & Lozano, 2012; Ponce & Lozano, 2011). This might be influenced by differences in the number specialists who work in specific field or the portion of basic research publications for each disease. A recent paper presented the bibliometrics for all the research studies under the term “MS” (Aleixandre‐Benavent et al., 2015). This study had a different viewpoint and methodological approach from our study, which might explain the discordance between the results of the two studies. We analyzed the top 100 cited articles on CIDD published during an unlimited time period, but other researchers included whole articles published in the *Multiple Sclerosis Journal* and articles under the term “MS” in the Web of Science database during 2003–2012.

Our research has some limitations that should be considered. First, as aforementioned, temporal bias is inevitable in a bibliometric analysis. Older articles can be more or less cited due to increased opportunities to be cited over time and the “obliteration by incorporation” phenomenon (Bohannon & Roberts, 1991; Garfield, 1987). There were limited Internet resources for the articles published before 1990. Similarly, the impact of recently published compelling articles can be underestimated due to an insufficient amount of time to accumulate citations. Use of other citation indices, such as average citations per year or time duration of high citations, may overcome this temporal bias, although results may still be influenced by the period of analysis. Second, several important articles might have been missed from our lists including articles published in journals in categories such as “immunology” or “pediatrics”. However, the proportion of articles in these categories was modest among the MS original research articles (Aleixandre‐Benavent et al., 2015). Third, incomplete citation bias might impact the results. Incomplete citations include various conscious or unconscious bias that arise from self‐citations, omissions, native language preponderance and the tendency to cite review articles or high impact factor journals (Ponce & Lozano, 2011; Seglen, 1997). Furthermore, there are other databases such as Scopus and Google Scholar that can be used for citation analysis. Selected search engines may have influenced the results (Kulkarni, Aziz, Shams, & Busse, 2009).

Here, we presented a detailed list of the top 100 cited articles for the topic CIDD and also separately propose citation classics for NMO using bibliometric methods. A strength of our research is that we included all types of articles published worldwide during an unrestricted time period. Although the citation rate does not directly represent the quality of the study, it is one marker used to recognize the importance of studies in the scientific community. We can trace scientific progress and identify seminal articles in a specific field by citation analysis.

## Conflict of Interest

All authors declare that they have no conflict of interest.
