# Supplementary material for: Citation classics in central nervous system inflammatory demyelinating disease
Source: Brain Behav. 2017 Apr 19;7(6):e00700. doi: 10.1002/brb3.700 (PMC5474706; doi:10.1002/brb3.700)
Supplement: Supplementary file 1 [file BRB3-7-e00700-s001.docx]

**Supplemental Table 1. Journals where the citation classics on central nervous system inflammatory demyelinating disease (A) and neuromyelitis optica (B) were published.**

| **Rank** | **Journal** | **No. of articles** |
| --- | --- | --- |
| 1. **CNS inflammatory demyelinating disease** | | |
| 1 | Brain | 24 |
| 2 | The New England Journal of Medicine | 21 |
| 3 | Neurology | 16 |
| 4 | Annals of Neurology | 15 |
| 5 | The Lancet | 12 |
| 6 | Archives of Neurology | 2 |
| 6 | Journal of Neuroscience | 2 |
| 6 | The Lancet Neurology | 2 |
| 6 | Nature Reviews Neuroscience | 2 |
| 10 | Annual Review of Neuroscience | 1 |
| 10 | Journal of the American Medical Association | 1 |
| 10 | Journal of the Neurological Sciences | 1 |
| 10 | Journal of Neurology, Neurosurgery and Psychiatry | 1 |
| 1. **Neuromyelitis optica** | | |
| 1 | Neurology | 15 |
| 2 | Brain | 7 |
| 3 | Archives of Neurology | 5 |
| 4 | Annals of Neurology | 3 |
| 5 | Journal of Neurology, Neurosurgery and Psychiatry | 2 |
| 5 | The Lancet Neurology | 2 |
| 7 | European Journal of Neurology | 1 |
| 7 | Journal of Neuroinflammation | 1 |
| 7 | Journal of the Neurological Sciences | 1 |
| 7 | Journal of Neurology | 1 |
| 7 | The Lancet | 1 |
| 7 | Nature Review Neurology | 1 |
| 7 | PLOS Medicine | 1 |

CNS, central nervous system**.**

**Supplemental Table 2. Authors of the top 100 cited articles on central nervous system inflammatory demyelinating disease. Only the authors who contributed three or more articles are presented.**

| **Author** | **No. of  citation classics** | **Types of author association according  to No. of citation classics** |
| --- | --- | --- |
| Miller DH | 15 | First (n=4), co- (n=8), corresponding (n=7) |
| Weinshenker BG | 13 | First (n=1), co- (n=10), corresponding (n=3) |
| Polman CH | 12 | First (n=3), co- (n=8), corresponding (n=4) |
| Lucchinetti CF | 12 | First (n=2), co- (n=9), corresponding (n=2) |
| Thompson AJ | 12 | co- (n=10), corresponding (n=2) |
| Barkhof F | 11 | First (n=1), co- (n=9), corresponding (n=2) |
| Lublin FD | 9 | First (n=1), co- (n=8), corresponding (n=1) |
| Kappos L | 8 | First (n=4), co- (n=4), corresponding (n=4) |
| Wingerchuk DM | 8 | First (n=3), co- (n=5), corresponding (n=2) |
| Hartung HP | 8 | First (n=1), co- (n=6), corresponding (n=2) |
| Confavreux C | 7 | First (n=3), co- (n=4), corresponding (n=3) |
| Reingold SC | 7 | co- (n=7) |
| Trapp BD | 6 | First (n=2), corresponding (n=6) |
| Comi G | 6 | First (n=2), co- (n=4), corresponding (n=2) |
| Pittock SJ | 6 | First (n=2), co- (n=4), corresponding (n=1) |
| Filippi M | 6 | First (n=1), co- (n=5), corresponding (n=1) |
| Lennon VA | 6 | First (n=1), co- (n=5), corresponding (n=1) |
| McDonald WI | 6 | First (n=1), co- (n=5), corresponding (n=1) |
| Rudick RA | 6 | First (n=1), co- (n=5), corresponding (n=1) |
| Lassmann H | 6 | co- (n=3), corresponding (n=3) |
| Brück W | 6 | co- (n=5), corresponding (n=1) |
| O'Connor P | 6 | co- (n=6) |
| Chang A | 5 | First (n=2), co- (n=3) |
| Compston A | 5 | First (n=1), co- (n=3), corresponding (n=1) |
| Antel JP | 5 | co- (n=5) |
| Edan G | 5 | co- (n=5) |
| Montalban X | 5 | co- (n=5) |
| Wolinsky JS | 5 | co- (n=5) |
| Peterson JW | 4 | First (n=1), co- (n=3) |
| Parisi JE | 4 | co- (n=4) |
| Coles AJ | 3 | First (n=2), corresponding (n=3) |
| Beck RW | 3 | First (n=2), co- (n=1), corresponding (n=2) |
| Munger KL | 3 | First (n=2), co- (n=1), corresponding (n=1) |
| Losseff NA | 3 | First (n=2), co- (n=1) |
| Ascherio A | 3 | First (n=1), co- (n=1), corresponding (n=2) |
| Cohen JA | 3 | First (n=1), co- (n=2), corresponding (n=1) |
| van Waesberghe JH | 3 | First (n=1), co- (n=2), corresponding (n=1) |
| Van Walderveen MA | 3 | First (n=1), co- (n=2), corresponding (n=1) |
| O'Riordan JI | 3 | First (n=1), co- (n=2) |
| Vollmer T | 3 | First (n=1), co- (n=2) |
| Arnold DL | 3 | co- (n=1), corresponding (n=2) |
| Francis GS | 3 | co- (n=3) |
| Hutchinson M | 3 | co- (n=3) |
| McFarland HF | 3 | co- (n=3) |
| Panzara MA | 3 | co- (n=3) |
| Paty DW | 3 | co- (n=3) |
| Radue EW | 3 | co- (n=3) |
| Rodriguez M | 3 | co- (n=3) |
| Sandberg-Wollheim M | 3 | co- (n=3) |
| Simon JH | 3 | co- (n=3) |
| Sørensen PS | 3 | co- (n=3) |
| Vukusic S | 3 | co- (n=3) |
| Waubant E | 3 | co- (n=3) |

**Supplemental Table 3. Authors of the citation classics on neuromyelitis optica. Only the authors who contributed three or more articles are presented.**

| **Author** | **No. of  citation classics** | **Types of author association according  to No. of citation classics** |
| --- | --- | --- |
| Weinshenker BG | 17 | First (n=1), co- (n=12), corresponding (n=5) |
| Wingerchuk DM | 14 | First (n=5), co- (n=9), corresponding (n=4) |
| Pittock SJ | 14 | First (n=3), co- (n=9), corresponding (n=3) |
| Lennon VA | 14 | First (n=1), co- (n=12), corresponding (n=2) |
| Lucchinetti CF | 14 | First (n=1), co- (n=12), corresponding (n=2) |
| Jacob S | 7 | First (n=1), co- (n=6), corresponding (n=1) |
| Vincent A | 6 | co- (n=4), corresponding (n=2) |
| Fujihara K | 6 | co- (n=6) |
| Misu T | 5 | First (n=2), co- (n=3), corresponding (n=2) |
| Nakashima I | 5 | First (n=1), co- (n=4), corresponding (n=1) |
| Itoyama Y | 5 | co- (n=5) |
| Waters PJ | 4 | First (n=2), co- (n=2), corresponding (n=1) |
| Takahashi T | 4 | First (n=1), co- (n=3), corresponding (n=1) |
| Palace J | 4 | co- (n=4) |
| Jacob A | 3 | First (n=1), co- (n=), corresponding (n=1) |
| Mandler RN | 3 | First (n=1), co- (n=2), corresponding (n=1) |
| Lassmann H | 3 | co- (n=2), corresponding (n=1) |
| Bergamaschi R | 3 | co- (n=3) |
| Hemmer B | 3 | co- (n=3) |
| Littleton E | 3 | co- (n=3) |
| Nakamura M | 3 | co- (n=3) |
| Reindl M | 3 | co- (n=3) |
| Sato S | 3 | co- (n=3) |
